# Butyrate attenuated fat gain through gut microbiota modulation in db/db mice following dapagliflozin treatment

**DOI:** 10.1038/s41598-019-56684-5

**Published:** 2019-12-30

**Authors:** Tae Jung Oh, Woo Jun Sul, Han Na Oh, Yun-Kyung Lee, Hye Li Lim, Sung Hee Choi, Kyong Soo Park, Hak Chul Jang

**Affiliations:** 1 Department of Internal Medicine, Seoul National University Bundang Hospital, Seongnam, Korea; 2 Department of Internal Medicine, Seoul National University College of Medicine, Seoul, Korea; 3 Department of Systems Biotechnology, Chung-Ang University, Anseong, Korea; 4 Department of Internal Medicine, Seoul National University Hospital, Seoul, Korea

**Keywords:** Endocrine system and metabolic diseases, Microbiome

## Abstract

We investigated the effect of a combination treatment with dapagliflozin (Dapa), a sodium-glucose cotransporter-2 inhibitor and butyrate on weight change in db/db mice. Six-week-old male db/db mice were assigned to four groups: vehicle with normal chow diet (NCD), Dapa with NCD, vehicle with 5% sodium butyrate-supplemented NCD (NaB), or Dapa with 5% NaB. After six weeks of treatment, faecal microbiota composition was analysed by sequencing 16S ribosomal RNA genes. In the vehicle with NaB and Dapa + NaB groups, body weight increase was attenuated, and amount of food intake decreased compared with the vehicle with the NCD group. The Dapa + NaB group gained the least total and abdominal fat from baseline. Intestinal microbiota of this group was characterized by a decrease of the Firmicutes to Bacteroidetes ratio, a decrease of *Adlercreutzia* and *Alistipes*, as well as an increase of *Streptococcus*. In addition, the proportion of *Adlercreutzia* and *Alistipes* showed a positive correlation with total fat gain, whereas *Streptococcus* showed a negative correlation. Inferred metagenome function revealed that tryptophan metabolism was upregulated by NaB treatment. We demonstrated a synergistic effect of Dapa and NaB treatment on adiposity reduction, and this phenomenon might be related to intestinal microbiota alteration.

## Introduction

Obesity is a major risk factor for diabetes and its complications, and increase in adiposity causes severe problems for diabetes management^1,2^. However, treatment of hyperglycaemia may induce weight gain due to diminished calorie loss through glycosuria^3^. In contrast to traditional oral hypoglycaemic agents, sodium-glucose cotransporter-2 (SGLT-2) inhibitors, a new class of oral medications, cause glycosuria and lead to pronounced calorie loss^4^. According to a recent meta-analysis, long-term treatment with SGLT-2 inhibitors led to a loss of 1–2 kg of body weight^5^. Therefore, SGLT-2 inhibitors are promising treatment options for diabetes as they reduce both hyperglycaemia and obesity.

In preclinical and clinical studies using SGLT-2 inhibitors, weight reduction was generally observed^5–7^, although the magnitude of weight reduction was less than expected^8^. One factor that is believed to prevent weight loss is compensatory hyperphagia^8^. Therefore, we need additional intervention strategies to control appetite and modulate adiposity when using SGLT-2 inhibitors. After SGLT-2 inhibitor treatment, a relative increase in glucagon level can induce lipolysis^9^. However, beta-hydroxybutyrate, the end-product of free fatty acid oxidation, inhibits lipolysis via negative feedback^10^. As a result, fat cells can reach equilibrium, and lipolysis might not be increased in the long term. Given this condition, administering a substance to induce lipolysis continuously may enhance the reduction in fat mass.

Butyrate is a short-chain fatty acid that is produced by fermentation of dietary fibre by gut microbiota^11^. In a human study, the abundance of butyrate-producing bacteria was increased in subjects with normal glucose tolerance compared with patients with diabetes^12^. An *in vitro* study showed that butyrate directly induced lipolysis in 3T3L-1 adipocytes^13^. Short-chain fatty acids including butyrate can be increased by the role of gut microbiota, which may then induce the production of gut-derived serotonin^14^, which plays a role in increasing lipolytic enzyme activity^15^. In mouse models of diet-induced obesity, butyrate attenuated weight gain and improved insulin resistance without decreasing food intake^16,17^. In other studies, appetite can be controlled by chronic administration of butyrate, which enhances intestinal gluconeogenesis^18^, modulates nutrient sensing, and augments gut hormones that inhibit the appetite centre in the hypothalamus^19^. Therefore, in this study, we hypothesized that butyrate treatment would induce lipolysis and control food intake in an db/db mouse model under dapagliflozin treatment. In addition, we investigated the alteration of gut microbiota composition and its plausible role in modulating host adiposity.

## Results

### Effects of dapagliflozin and butyrate on body weight, food intake, and glucose metabolism in db/db mice

**Figure 1:**
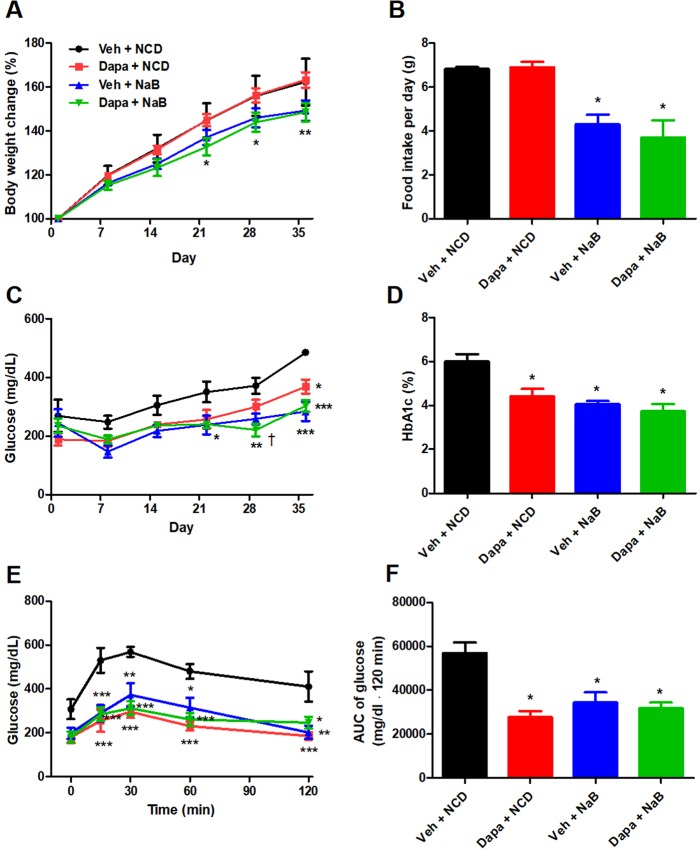
Serial percent changes of body weight (**A**), food intake during 24 h (**B**), serial levels of random glucose (**C**), HbA1c levels after six weeks of treatment (**D**), glucose profiles during oral glucose tolerance tests (**E**), and area under the curve of glucose levels (**F**). Data are represented as mean ± standard error. *P < 0.05, **P < 0.01, *** P < 0.001 vs. Veh + NCD, ^†^*P* < 0.05 vs. Dapa + NCD.

The percent changes of body weight were not different between the Veh group and the Dapagliflozin (Dapa) group. However, it was significantly lower in the Dapa + sodium butyrate (NaB) group than in the Vehicle (Veh) group (Fig. 1A). The average food intake during 24 h during 6-week observation was comparable between the Veh group and the Dapa group, but it was decreased in the NaB and Dapa + NaB groups compared with the Veh group (Fig. 1B). Serial random glucose levels were lower in the Dapa + NaB group (starting from week three) and in the Dapa and NaB groups (starting from week five) than in the Veh group (Fig. 1C). At week four, glucose levels decreased more in the Dapa + NaB group than in the Dapa group. We also detected a decrease of HbA1c levels in these three treatment groups compared with the Veh group (Fig. 1D). During oral glucose tolerance tests (OGTTs), glucose excursion in these groups was markedly decreased compared with the Veh group (Fig. 1E,F).

### Fat mass change, and fat and liver histology

**Figure 2:**
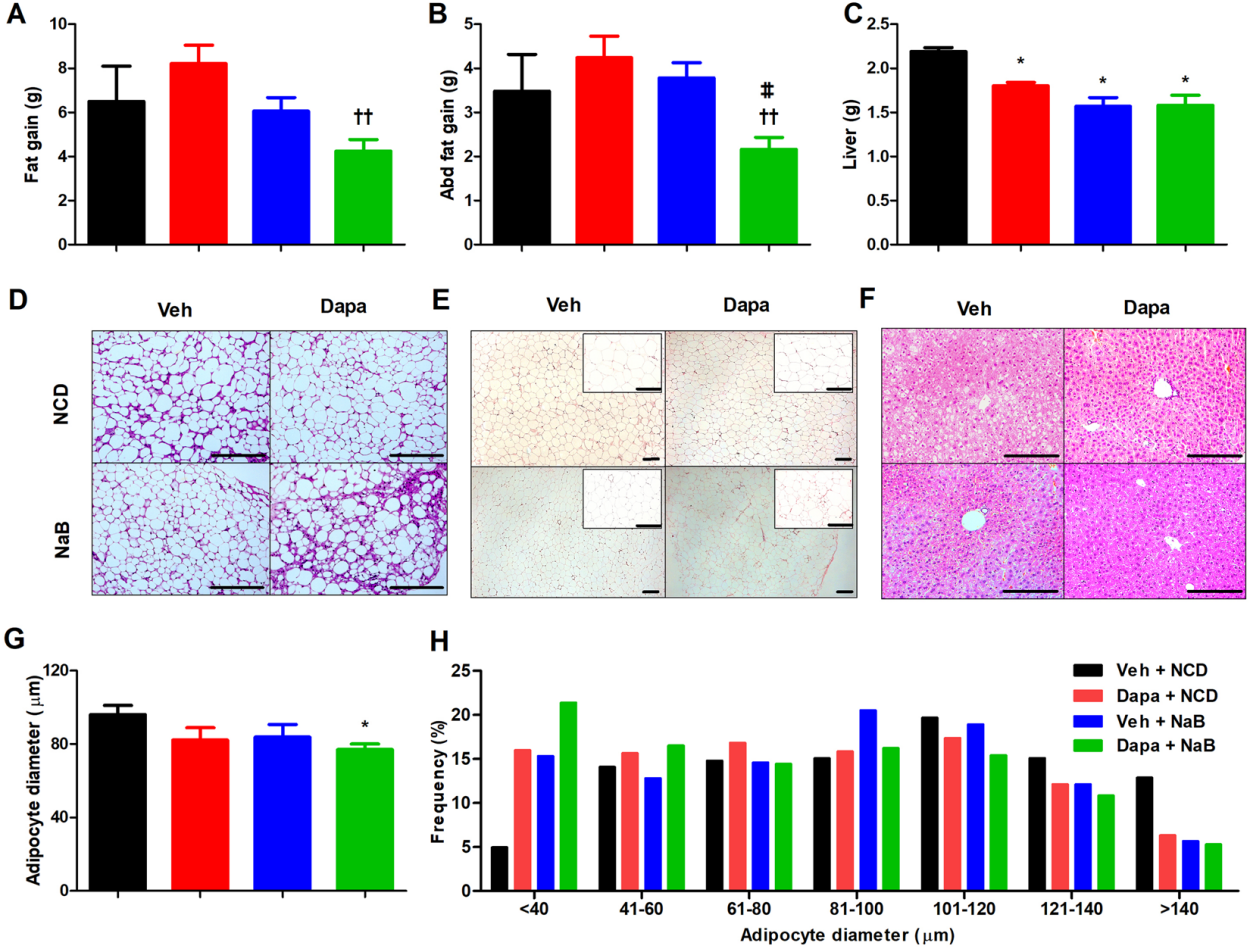
Fat gain from baseline (**A**), abdominal fat gain from baseline (**B**), liver weight (**C**), H&E staining of brown adipose tissue. Scale bars, 200 µm (**D**), inguinal white adipose tissue. Scale bars, 200 µm. Images on the right are high-magnification images (**E**), and liver. Scale bars, 200 µm (**F**), adipocyte diameter (**G**), and adipocyte size distribution of inguinal white adipose tissue (**H**). Data are represented as mean ± standard error. *P < 0.05 vs. Veh + NCD, ^††^*P* < 0.01 vs. Dapa + NCD, ^‡‡^*P* < 0.001 vs. Veh + NaB.

Both fat and abdominal fat gain from baseline was not decreased in the Dapa group compared to the Veh group. In the Dapa + NaB group, fat gain was more attenuated than the Dapa group (Fig. 2A). Furthermore, abdominal fat gain was also lower in the Dapa + NaB group than in the Dapa or NaB groups (Fig. 2B). Multilocular lipid droplets of brown adipose tissue (BAT) were found by haematoxylin and eosin (H&E) staining in the Dapa + NaB group (Fig. 2D). The mean adipocyte diameter in inguinal white adipose tissue (iWAT) was decreased in the Dapa + NaB group compared with the Veh group (Fig. 2E,G), and small-sized adipocytes in iWAT tended to be increased in the Dapa + NaB group (Fig. 2H). Liver weight was decreased, and fat accumulation in the liver was attenuated in the Dapa, NaB, and Dapa + NaB groups than the Veh group (Fig. 2C,F).

### Plasma levels of metabolic hormones and lipolysis-related gene expression in iWAT

**Figure 3:**
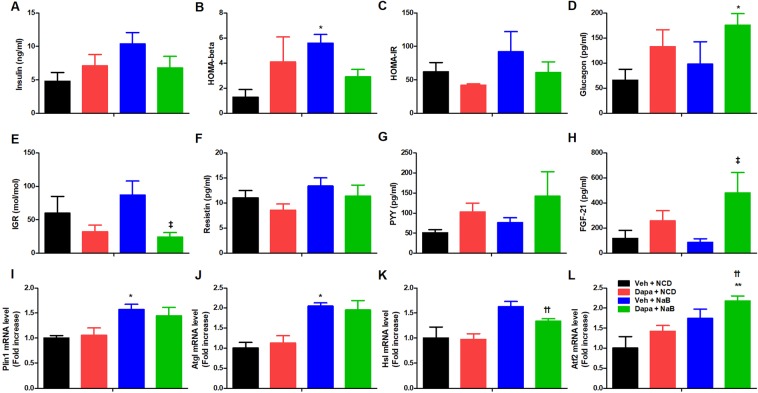
Plasma levels of insulin **(A**), HOMA-beta (**B**), HOMA-IR (**C**), plasma levels of glucagon (**D**), insulin to glucagon ratio (**E**), plasma levels of resistin (**F**), peptide YY (**G**), and FGF-21 (**H**), mRNA expression of perilipin 1 (**I**), adipose triglyceride lipase (**J**), hormone-sensitive lipase (**K**), and activating transcription factor 2 (**L**) in inguinal white adipose tissue. Data are represented as mean ± standard error. *P < 0.05 vs. Veh + NCD, ^††^*P* < 0.01 vs. Dapa + NCD, ^‡^*P* < 0.05 vs. Veh + NaB.

Fasting insulin levels tended to be higher in the NaB group than in the Veh group (10.4 ± 1.7 vs. 4.8 ± 1.3 ng/ml, *P* = 0.057) (Fig. 3A). Accordingly, homeostatic model assessment of beta cell function (HOMA-beta) was higher in the NaB group than in the Veh group (Fig. 3B), but HOMA-insulin resistance (HOMA-IR) was comparable between groups (Fig. 3C). Glucagon levels were higher in the Dapa + NaB group than in the Veh group (Fig. 3D). The insulin to glucagon ratio (IGR) was decreased in the Dapa + NaB group compared with the NaB group (24.1 ± 6.6 vs. 87.3 ± 21.4 mol/mol, *P = *0.032) (Fig. 3E). There was no significant difference in plasma levels of resistin and peptide YY (PYY) between groups (Fig. 3F,G). The fibroblast growth factor 21 (FGF-21) levels were significantly increased in the Dapa + NaB group compared with the NaB group (85.0 ± 30.3 vs. 481.4 ± 162.6 pg/ml, *P* = 0.016) (Fig. 3H). There was higher mRNA expression of perilipin 1 (*Plin1*), and adipose triglyceride lipase (*Atgl*) in iWAT of the NaB group compared with the Veh group (Fig. 3I,J). The mRNA expression of hormone-sensitive lipase (*Hsl*) in iWAT was increased in the Dapa + NaB group than the Dapa group (Fig. 3K). The activating transcription factor 2 (*Atf2*) in iWAT derived from Dapa + NaB group was higher compared with the Veh and the Dapa groups (Fig. 3I–L).

### Tryptophan hydroxylase-1 and tight junction protein gene expression in intestine

**Figure 4:**
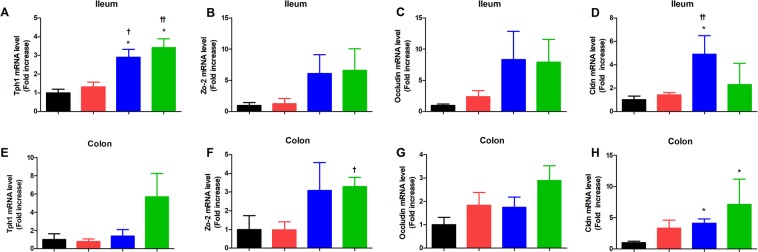
The mRNA expression of tryptophan hydroxylase-1 (*Thp-1*), *Zo-2*, *occludin*, and Claudin (*Cldn*) in ileum (**A–D**) and colon (**E–H**). *P < 0.05 vs. Veh + NCD, ^†^*P* < 0.05, ^††^*P* < 0.01 vs. Dapa + NCD.

The mRNA expression of tryptophan hydroxylase-1 (*Thp-1*), a rate liming enzyme for serotonin synthesis^14^ was higher in ileum of the NaB and Dapa + NaB groups than Veh or Dapa groups (Fig. 4A). In colon tissue, the mRNA expression of *Thp-1* showed the increased tendency in the Dapa + NaB group compared to other groups (Fig. 4E). In addition, Claudin (*Cldn*) was significantly higher in the NaB group than the Veh group or the Dapa group (Fig. 4D), but mRNA levels of other tight junction protein did not show any significant difference between groups (Fig. 4B,C). In colon, *Zo-2* of Dapa + NaB group was higher than the Dapa group, and *Cldn* of NaB and Dapa + NaB groups was higher than the Veh group (Fig. 4F,H). There was no difference in *Occludin* mRNA level between groups (Fig. 4G).

### Distinct gut microbiota composition following NCD versus NaB treatment

**Figure 5:**
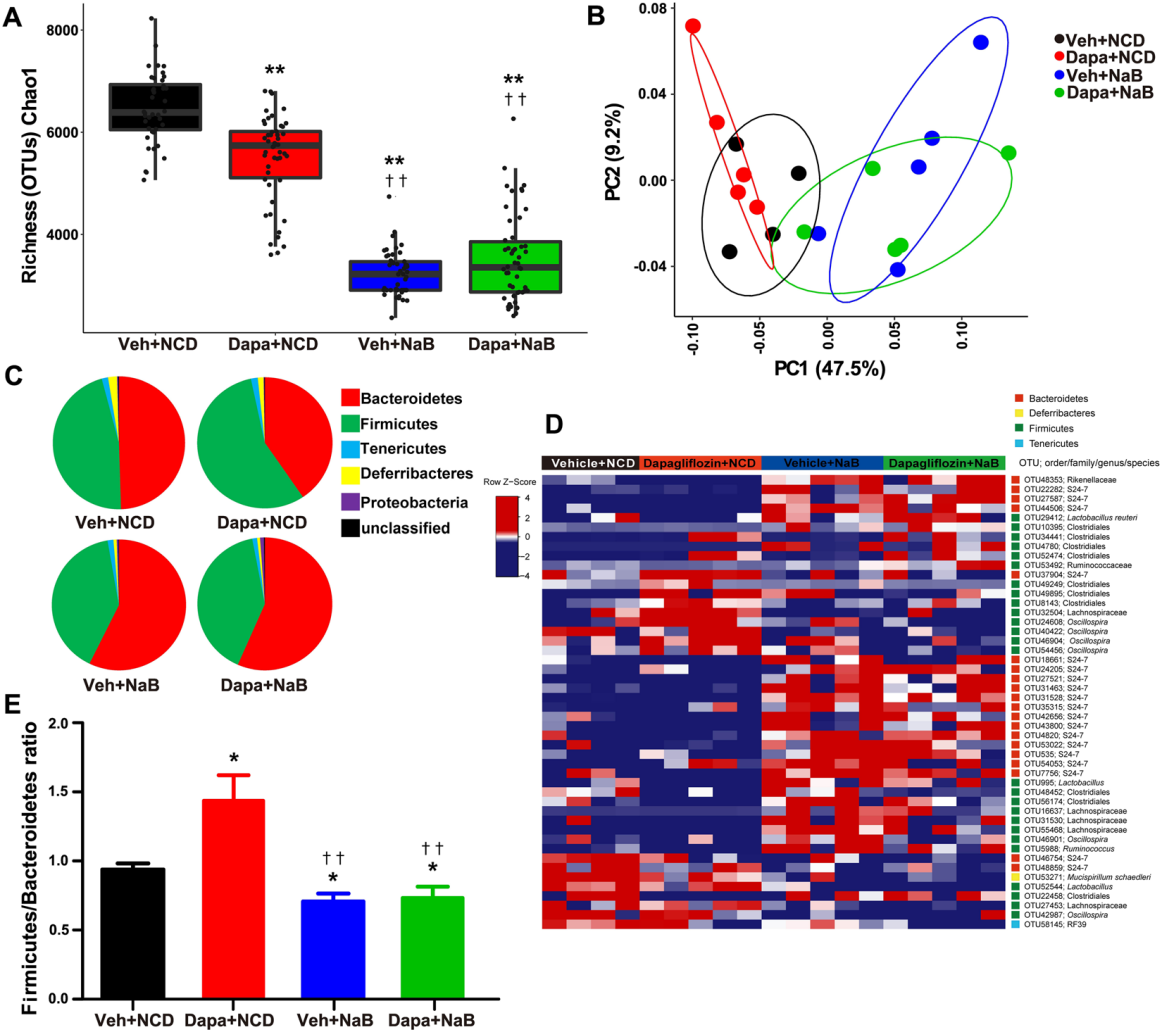
α-Diversity (Chao1) comparison of the gut microbiota of the four treatment groups (**A**), PCoA plot of weighted UniFrac distances based on 97% similar OTUs across the different groups (**B**), composition of abundant bacterial phyla in the gut microbiota (**C**), relative ratio of Firmicutes to Bacteroidetes (**E**), and heat map for significantly different OTUs determined by LEfSe analysis (LDA >3.0) according to treatment groups (**D**).

To identify the intestinal microbiota composition in the mice of the four treatment groups, we collected mice stool samples and conducted 16S ribosomal RNA gene sequencing using the Illumina MiSeq platform. From the stool samples, a total of 2,366,458 merged sequences and 58,624 operational taxonomic units (OTUs) were obtained. We found a significant decrease of microbial diversity in the NaB-treated group, regardless of additional Dapa treatment (Fig. 5A). Principal co-ordinate analysis (PCoA) analysis with weighted UniFrac distances confirmed the heterogeneity of gut microbiota among the four treatment groups. A clear separation between gut microbial communities, driven by NaB, was observed (Fig. 5B). Analysis at the phylum level revealed that the relative abundance of Bacteroidetes was decreased in the Dapa group, but it was increased following NaB treatment (Fig. 5C). The relative ratio of Firmicutes to Bacteroidetes was increased in the Dapa group compared with the Veh group, but it was decreased in the NaB and NaB + Dapa groups compared with the Veh or Dapa groups (Fig. 5E). Furthermore, the heat map shows significantly different OTUs in the four treatment groups, with a logarithmic linear discriminant analysis (LDA) score of ≥3.0 as determined by linear discriminant analysis effect size (LEfSe) analysis (Fig. 5D). The relative abundance of OTUs was well separated by the presence of NaB.

### Microbiota signature changes in relation to body fat change

**Figure 6:**
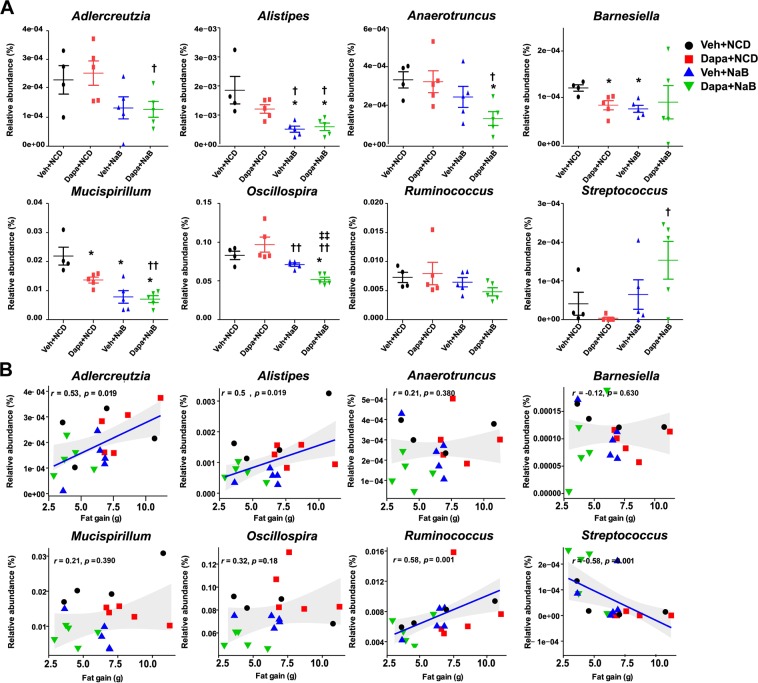
Relative abundance of eight selected bacterial genera (**A**), and Spearman correlation analysis between fat gain and relative abundance of bacterial genus (**B**). *P < 0.05 vs. Veh + NCD, ^†^*P* < 0.05, ^††^*P* < 0.01 vs. Dapa + NCD, ^‡‡^*P* < 0.001 vs. Veh + NaB.

At the genus level, the mice intestinal microbiota differed in the Dapa + NaB group compared with the Veh group. The relative abundance of the genera *Alistipes*, *Anaerotruncus*, *Mucispirillum*, and *Oscillospira* was markedly decreased in the Dapa + NaB group compared with the Veh group (Fig. 6A). Compared with the Dapa group, *Adlercreutzia*, *Alistipes*, *Anaerotruncus*, *Mucispirillum*, and *Oscillospira* abundance was also decreased in the Dapa + NaB group. However, *Streptococcus* abundance was significantly increased in the Dapa + NaB group compared with the Dapa group. Interestingly, the amount of fat gain was positively correlated with relative abundance of *Adlercreutzia* and *Alistipes*, but negatively correlated with *Streptococcus* (Fig. 6B).

### Characterization of gut microbiota functionality

**Figure 7:**
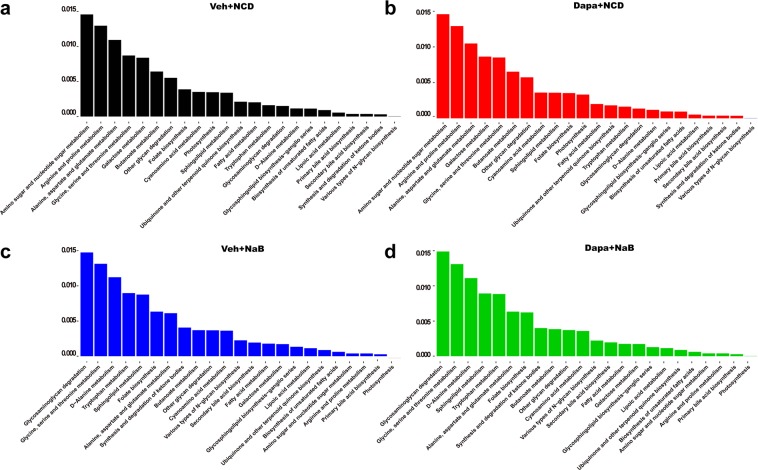
Bar graph of functional KEGG pathways inferred from metagenome analysis according to treatment groups.

Metagenome functionality analysis, inferred from the 16S rRNA gene sequences, revealed that amino and nucleotide sugar metabolism, arginine and proline metabolism, and alanine, aspartate, and glutamate metabolism were the top three upregulated pathways in the Veh and Dapa groups (Fig. 7a,b). However, glycosaminoglycan degradation, glycine, serine, and threonine metabolism, and D-alanine metabolism were commonly upregulated in the NaB and Dapa + NaB groups (Fig. 7c,d). Interestingly, tryptophan metabolism was commonly upregulated in the NaB and Dapa + NaB groups but it was not upregulated in the other groups.

## Discussion

Chronic treatment with dapagliflozin did not reduce body weight and fat gain in db/db mice. However, cotreatment with butyrate and dapagliflozin attenuated increasing adiposity in this mouse model. In addition, butyrate itself can modulate food intake and hyperglycaemia. The combination treatment of butyrate and dapagliflozin increased intestinal serotonin producing enzyme and may have increased lipolysis. The anti-obesity effects of butyrate were highly correlated with alterations of gut microbiota composition, especially the decrease of *Adlercreutzia* and *Alistipes*, and the increase of *Streptococcus*.

The main mechanism of butyrate that modulated adiposity was the decrease of food intake. Interestingly, even though there was no difference in food intake between butyrate monotherapy and the combination treatment with dapagliflozin and butyrate, abdominal fat gain was markedly decreased with the combination treatment compared with butyrate monotherapy. However, the effect of combination treatment on the abdominal fat was not solely related with gut microbiota because there was no big difference in the microbiota composition between the Veh + NaB and Dapa + NaB groups. The main contributor for microbiota change was butyrate. Therefore, we did not see further microbiota change after the dapagliflozin add-on therapy to butyrate compared with butyrate monotherapy. There might be other contributor to regional fat tissue modulation when this combination treatment is used. Histologic analysis revealed decreased diameter of adipocytes and increased proportions of small adipocytes in the combination treatment group. There was evidence that butyrate directly induced fat lipolysis^13^, and the relative increase of glucagon after SGLT-2 inhibitor treatment could induce breakdown of fat and lead to the use of fatty acids as energy source^20,21^. Therefore, this combination treatment might be efficient in inducing fat lipolysis. In the same context, we observed higher plasma levels of FGF-21, a key regulator of lipolysis^22^, and lower levels of IGR in the Dapa + NaB group compared with the NaB group. A recent longitudinal observational study also showed that abnormal subcutaneous fat-cell lipolysis is related to long-term weight gain^23^. Interestingly, we observed higher *Atf2* mRNA levels in iWAT of the Dapa + NaB group compared with the Veh and Dapa groups. ATF2 has been revealed to bind at the *Fgf21* gene promoter region and induce *Fgf21* gene transcription^24^. Therefore, efficient fat lipolysis after cotreatment with butyrate and dapagliflozin might be regulated by the ATF2–FGF-21 axis. Furthermore, the increase of glucagon and the decrease of IGR could be related with increase of thermogenesis^25^ in the combination treatment. In summary, combination treatment reduced abdominal fat much more than single treatments via both the alteration of microbiota and induction of glucagon action, but this hormonal change was not directly associated with microbiota composition.

Butyrate reduced hyperglycemia as much as dapagliflozin did. Butyrate reduced food intake and dapagliflozin induced glycosuria; these mechanisms were distinct from each other. However, we unfortunately did not see a further decrease of hyperglycemia in the combination treatment. In our experiment, we treated butyrate in db/db mice at an early stage of obesity and diabetes from when they were six weeks old. The db/db mice develop hyperphagia-induced obesity and diabetes^26^. Therefore, the initiation timing of butyrate might be a critical factor to prevent weight gain and severe hyperglycaemia through reducing food intake. Under butyrate, the effect of dapagliflozin might not be apparent in terms of glucose-lowering ability. However, human obesity and diabetes are generally developed by multifactorial conditions rather than just by hyperphagia. From this clinical perspective, it is necessary to test butyrate as a supplement with other anti-diabetic or anti-obesity medication in human studies.

Butyrate is normally produced in high concentration by bacterial fermentation in the distal intestine^11^. However, we administered NaB mixed into the diet as a supplement. Therefore, butyrate was primarily absorbed via the upper intestine and was not likely to stimulate the distal part of the gut where enteroendocrine cells are abundantly expressed. In addition, plasma PYY levels were not increased in the NaB-treated groups. A previous study also found no increase in distal gut hormones after oral butyrate administration, which contradicts findings reported using a high-fibre diet^27^. Even though there was the possibility that butyrate did not reach the distal intestine, this intervention altered overall gut microbiota composition. The relative abundance of Bacteroidetes was increased in the NaB-treated groups, and that of Firmicutes was decreased. The decreased ratio of Firmicutes to Bacteroidetes was also observed in a previous study that tested the effects of butyrate administered by oral gavage in db/db mice^28^. That study showed anti-inflammatory effects of butyrate and its protective role in gut epithelial barrier integrity. Butyrate has a possible role in the modulation of intestinal immunity. Butyrate treatment activates regulatory T cells^29^ and inhibits lipopolysaccharide-induced-proinflammatory mediators produced by intestinal macrophages^30^. Another possible explanation is that the reduction in food intake *per se* may modulate the microbiota composition. One study found that a low-calorie diet changes the gut microbiota composition^31^. In our experiments, the expression of the gene encoding intestinal tight junction protein was increased after butyrate treatment. The loss of intestinal integrity could induce low-grade systemic inflammation and finally increase metabolic disorders^32^. In addition, tryptophan metabolism was upregulated by butyrate treatment, and this upregulation was related to the induction of intestinal serotonin production^14^. Our findings suggest that butyrate can modulate both gut health and function by changing the gut microbial composition, which may alter aspects of host metabolism such as lipolysis activity.

A previous study using a human-like, diet-induced mice model of obesity also reported microbiota alteration in the faeces after butyrate treatment, and it demonstrated that oral administration of butyrate alone can—in contrast to intravenous administration—suppress food intake^33^. However, food intake was not reduced after butyrate treatment in vagotomized mice^33^. Therefore, the metabolic effects of butyrate might be driven via the intestine rather than via direct effects on the central nervous system. If we had used butyrate and dapagliflozin in vagotomized mice model or performed pair-fed experiments, we would have a clear idea about a direct effect of this combination in adipose tissue excluding the effect of calorie restriction.

It is controversial whether the ratio of Firmicutes to Bacteriodetes is relates with obesity^34^ and the role of microbial diversity in obesity is also controversial^35^. Obesity has been reported to be related to reduced microbial diversity^36^, but our result and those of others^37,38^ show reduced microbial diversity even after improvements in host metabolism. It is necessary to consider the altered composition of microbiota in more specific levels and its crosstalk with host. In our study, we showed that *Streptococcus*, belonging to the Firmicutes phylum, was negatively correlated with fat mass gain, whereas the abundance of *Alistipes*, within the Bacteroidetes phylum, correlated positively with fat mass gain. A previous study also showed similar association between *Alistipes* and body fat and inflammatory cytokines^39,40^. In contrast, using the data from 1,914 Chinese adults, Zeng *et al*. reported that *Alistipes* was negatively associated with metabolic abnormalities such as obesity, hypertension, and dyslipidemia^41^. Therefore, ethnicity or dietary habits might influence the abundance of *Alistipes*. In fact, dietary intervention in human studies rapidly altered the microbial composition; for example, *Alistipes* was increased after an animal-based diet^42^. In an experimental model, *Alistipes* was also increased after a high fat diet^43^. In summary, there were discrepancies in the results of the alteration of *Alistipes* according to host metabolic status. However, *Alistipes* might be positively related with obesity, at least in the well-controlled animal experiments^39,43^. Proof of a direct effect of *Alistipes* on adiposity will require studies of faecal transplantation or therapeutic administration. In the case of *Adlercreutzia*, obesity was positively correlated with the high abundance of *Adlercreutzia* in human studies^44,45^.

The current study has several limitations. First, we did not compare the metabolic effects with pair-fed groups. However, dapagliflozin monotherapy did not induce hyperphagia as initially expected. Therefore, it is not necessary to match the food intake of the Dapa and Veh groups. Second, we cannot demonstrate a role of gut microbiota in reducing adiposity, because we did not perform faecal transplantation. Third, there were no data on energy expenditure, which could provide insight into the changes of metabolic rates after intervention. Fourth, we used genetically obese mouse model. Therefore, there is a gap between current observation and human biology. Butyrate directly increases leptin expression in adipocytes^46^ but reduces systemic leptin level *in vivo* after chronic treatment^47^; the latter effect may relate to the reduction in whole-body fat mass. Therefore, the systemic leptin level may increase in short-term experiments but may decrease after long-term treatment. However, there was no published evidence that butyrate can recover leptin resistance. In our experiments, we excluded an effect of leptin by using leptin receptor-knockout mice. Butyrate supplementation combined with an SGLT-2 inhibitor should be tested in a high-fat diet-induced obesity mouse model. Obese people with type 2 diabetes may also be a good candidate group for studies to confirm the anti-obesity effect of the combined treatment of butyrate and an SGLT-2 inhibitor. In contrast to GLP-1 receptor agonists, SGLT-2 inhibitors are the only oral anti-diabetic medication shown to reduce body weight, and they have potential for wide use. Further research is needed to study the effects of other supplements to boost the anti-obesity effect of SGLT-2 inhibitors. Butyrate may be one candidate for application in human studies.

In conclusion, butyrate supplement reduced fat and, in combination with dapagliflozin, further modulated abdominal obesity. Alterations in gut microbiota composition and functionality were related to the anti-obesity effects of butyrate.

## Methods

### Study animals and treatment

Five-week-old male db/db mice (Envigo, Huntingdon, Cambridgeshire, UK) were housed in a light- and temperature-controlled facility. To minimize the inter-group difference at baseline, after one week of adaptation, the mice were assigned to four groups according to their body weight and random glucose levels: (1) veh with chow diet (LabDiet 5L79, LabDiet, St. Louis, MO, USA) (Veh group); (2) Dapa (U Chem Co., Anyang-si, Gyeonggi-do, Korea) with NCD (Dapa group); (3) veh with 5% NaB-supplement NCD (303410, Sigma-Aldrich, St. Louis, MO, USA) (NaB group); and (4) Dapa with 5% NaB-supplement NCD (Dapa + NaB group). Dapagliflozin (1 mg/kg) or veh (normal saline) was administered daily via oral gavage for six weeks. The dosages of butyrate and dapagliflozin were chosen based on previous studies of their anti-obesity^16,17^ and anti-diabetic effects^48,49^. All animals were allowed food and water *ad libitum*. Whole body and abdominal fat were measured using dual-energy X-ray absorptiometry (InAlyzer, Medikors, Seoul, Korea) under general anesthesia with 2% isoflurane. Animal experiments were approved by the Institutional Animal Care and Use Committee of Seoul National University Bundang Hospital, South Korea (No. BA 1511-188/072-04). All animal experiments were performed in accordance with the approved guidelines on the use of laboratory animal of Seoul National University and Seoul National University Bundang Hospital.

### Oral glucose tolerance test

After six weeks of treatment, OGTTs were performed after 15 h of fasting. Baseline blood glucose levels were measured and then glucose (0.5 g/kg body weight) was given orally as a 20% solution. Serial glucose measurements were performed at 15, 30, 60, and 120 min using an Accu-Chek Performa glucometer (Roche Diagnostics, Mannheim, Germany).

### Biochemical analysis

Fasting blood samples were obtained by cardiac puncture in mice anesthetized with Zoletil 50 (zolazepam plus tiletamine, Virbac Laboratories, Carros, France). Blood was collected into EDTA tubes containing aprotinin 500 KIU/mL. The tubes were placed on ice immediately and centrifuged at 1500 g for 20 min at 4 °C. Plasma was stored at –80 °C until used for biochemical analysis. HbA1c levels were measured by high performance liquid chromatography (Arkray Inc., Kyoto, Japan) according to the National Glycohemoglobin Standardization Program standard at Seoul National University Bundang Hospital’s central laboratory. Insulin, glucagon, resistin, and PYY were measured using a Milliplex Mouse Metabolic Hormone Panel (MMHMAG-44K, Merck Millipore, Billerica, MA, USA). HOMA-IR and HOMA-beta were calculated using fasting glucose and insulin levels^50^. The IGR was assessed by a previously reported method^21^. Plasma FGF-21 concentrations were measured using an ELISA kit (Biovender, Brno, Czech Republic).

### RNA isolation and quantitative real-time PCR

Total RNA was extracted from iWAT, distal ileum, and colon samples using TRIzol (Ambion, CA, USA). For quantitative real-time PCR analysis, we followed the previously published method^51^. The 3 μg of total RNA was reverse-transcribed using a High Capacity cDNA Reverse Transcription Kit (Thermo Fisher Scientific, Waltham, USA). SYBR Green reactions using the SYBR Green PCR Master mix (Enzynomics, Daejeon, Korea) were assembled along with 10 pM primers according to the manufacturer’s instructions and were performed using the Applied Biosystems ViiA7 system (Thermo Fisher Scientific). Relative mRNA levels were calculated using the comparative CT method and normalized to *cyclophilin* mRNA for iWAT and *GAPDH* mRNA for distal ileum and colon. mRNA expression levels are displayed relative to Veh control mRNA as specified for each experiment. The sequences of all primers used are listed in Supplementary Table S1.

### Histology of adipose tissue

Mice were sacrificed by cardiac puncture under anaesthesia. The iWAT from bilateral inguinal area, BAT from the interscapular area, and the liver were collected and fixed in 10% neutral formaldehyde. Remaining tissue was frozen immediately in liquid nitrogen. Paraffin sections were made and stained with H&E. Slides were scanned with a photomicroscope (Axioskop 40, Carl Zeiss, Germany). We manually drew the maximal diagonal length of each adipocyte in calculating adipocyte diameters.

### Faecal DNA extraction and sequencing

Genomic DNA was extracted from approximately 0.25 g of each mouse stool sample using the QIAamp PowerFecal Kit (Qiagen, Hilden, Germany) and was quantified using a NanoDrop 2000 spectrophotometer (Thermo Fisher Scientific). Amplification of the V4–V5 region of the 16S rRNA gene was performed by PCR using the bacterial-specific primers, 518 F (5′-TCG TCG GCA GCG TCA GAT GTG TAT AAG AGA CAG CCA GCA GCY GCG GTA AN-3′) and 927 R (5′-GTC TCG TGG GCT CGG AGA TGT GTA TAA GAG ACA GCC GTC AAT TCN TTT RAG T-3′). The conditions of thermal cycling were 95 °C for 3 min for denaturation, 25 cycles of amplification (95 °C for 30 s, 55 °C for 30 s, and 72 °C for 30 s), and a final extension at 72 °C for 5 min. The barcoded sequences (Illumina Nextera XT Index Kit v2, Illumina, San Diego, CA, USA), were incorporated using index PCR primers under the same conditions. Each sample was quantified and qualified using a NanoDrop spectrophotometer, and samples were pooled for MiSeq sequencing performed by Macrogen Corp (Illumina, Seoul, Korea). The raw sequence data used in this study were uploaded to SRA with the accession number SRR8371999 to SRR8372017.

### Bacterial phylogenetic analysis

The raw sequencing data were processed and merged using a Python script, *iu-merg-pairs* (REF) with default options (minimum overlap region size: 15, minimum quality score: 15)^52^ and the merged paired-end sequences were analysed using the QIIME (Quantitative Insights into Microbial Ecology) pipeline (version 1.9.1)^53^. Merged sequences with 97% similarity were clustered into OTUs by UCLUST^54^, and representative sequences of OTUs were used to assign bacterial taxa from the RDP database^55^. Sequences assigned to mitochondria and chloroplasts, which are considered eukaryotic DNA sequences, were discarded. The Chao1 index was used to calculate α-diversity, weighted UniFrac distance matrices were used to calculate β-diversity, and PCoA with QIIME script (alpha_diversity.py, beta_diversity.py) was used to visualize results. Statistical significance of treatment differences was determined by analysis of similarities (ANOSIM) with 999 permutations.

### Functional prediction

To predict inferred metagenome functions from the 16S rRNA gene sequences, PICRUSt analysis was used^56^. OTUs with 97% similarity were selected and taxonomically assigned. OTUs corresponding to chloroplasts and mitochondria were removed from the OTUs normalized by copy number. KEGG pathways were analysed at level three and normalized to the total of predicted pathways of each sample. Among the 328 pathways, 93 that are considered to be associated with metabolism were selected.

### Statistical analysis

Data are presented as mean ± SEM. Time course differences were analysed with two-way repeated measures analysis of variance (ANOVA) with Bonferroni *post hoc* analysis. Two-group or four-group comparisons were conducted using a *t* test and one-way ANOVA. Area under the curve was calculated using the trapezoidal rule. To compare the specific OTU differences across Dapa and NaB treatment groups, LEfSe analysis was performed^57^. The threshold for the non-parametric factorial Kruskal–Wallis sum-rank test was α = 0.05. The threshold for the logarithmic LDA score was 3.0. Spearman’s correlation analysis was applied to test the correlation between total fat gain and relative abundance of genera that were significantly different in mice faecal microbiota in the four treatment groups. Statistical analysis was performed using Prism (v. 5.0; GraphPad, San Diego, CA, USA) and R software (v. 3.3.2; R Foundation, Vienna, Austria). To find statistically significant functions across the butyrate treatment, t-test was conducted and local FDR that calculated with respect to a single value was estimated^58^. *P* < 0.05 was considered significant.

## Supplementary information

Supplementary information.

## Data Availability

The data generated during the current study are available from the corresponding author on reasonable request.
