# Supplementary material for: Butyrate attenuated fat gain through gut microbiota modulation in db/db mice following dapagliflozin treatment
Source: Sci Rep. 2019 Dec 30;9:20300. doi: 10.1038/s41598-019-56684-5 (PMC6937275; doi:10.1038/s41598-019-56684-5)
Supplement: Supplementary file 1 — Supplementary information. [file 41598_2019_56684_MOESM1_ESM.docx]

**Butyrate attenuated fat gain through gut microbiota modulation in db/db mice following dapagliflozin treatment**

Tae Jung Oh^1,2^*, Woo Jun Sul^3^*, Han Na Oh^3^, Yun-Kyung Lee^1^, Hye Li Lim^1^, Sung Hee Choi^1,2^, Kyong Soo Park^2,4^, Hak Chul Jang^1,2^

^1^Department of Internal Medicine, Seoul National University Bundang Hospital, Seongnam, Korea

^2^Department of Internal Medicine, Seoul National University College of Medicine, Seoul, Korea

^3^Department of Systems Biotechnology, Chung-Ang University, Anseong, Korea

^4^Department of Internal Medicine, Seoul National University Hospital, Seoul, Korea

Correspondence should be addressed to

Tae Jung Oh, MD, PhD

Department of Internal Medicine, Seoul National University College of Medicine and Seoul National University Bundang Hospital

300 Gumi-dong, Bundang-gu, Seongnam-city, Korea (Postal code: 13620)

Phone: 82-31-787-7078, Fax: 82-31-787-4050, E-mail: [ohtjmd@gmail.com](mailto:ohtjmd@gmail.com)

OR

Woo Jun Sul, PhD

Department of Systems Biotechnology, Chung-Ang University, Anseong, Korea

4726, Daedeok-myeon, Seodong-daero, Anseong-city, Gyeonggi-do, Korea (Postal code: 17546)

Phone: +82-31-670-4707, Fax: +82-31-670-3108, E-mail: [sulwj@cau.ac.kr](mailto:sulwj@cau.ac.kr)

**Word count:** 198 in abstract and 4,424 in manuscript

**Number of figures:** 7 figures (1 supplementary table)

| **Table S1. Sequence of primers for quantitative real-time PCR** | | |
| --- | --- | --- |
| Target gene | Forward Primer | Reverse Primer |
| *Plin1* | GATCGCCTCTGAACTGAAGG | CTTCTCGATGCTTCCCAGAG |
| *Atgl* | TCCGAGAGATGTGCAAACAG | CTCCAGCGGCAGAGTATAGG |
| *Hsl* | GCGCTGGAGGAGTGTTTTT | CCGCTCTCCAGTTGAACC |
| *Atf2* | CCATCGCCCTGCAATGTTTT | TCTGGCAACCACATCACACT |
| *Tph1* | TAGAAGTATGTCCACGGGCCT | GGACGGATGGAAAACCCAGT |
| *Zo2* | TCCTCCACTGCAGCTTGTAGTTC | TCACGGTGTACTGCTCCCAT |
| *Occludin* | GTGAATGGGTCACCGAGGG | AGATAAGCGAACCTGCCGAG |
| *Cldn* | CCACCATTGGCATGAAGTGC | AGAGGTTGTTTTCCGGGGAC |

Plin1, perilipin 1; Atgl, adipose triglyceride lipase; Hsl, hormone-sensitive lipase; Atf2, activating transcription factor 2; TPH1, tryptophan hydroxylase-1; Cldn, claudin
